# Understanding factors influencing healthcare workers’ intention towards the COVID-19 vaccine

**DOI:** 10.1371/journal.pone.0286794

**Published:** 2023-07-27

**Authors:** Zhuyun Xie, Sikandar Ali Qalati, Mónica Lorena Sánchez Limón, Mohammad Ali Bait Ali Sulaiman, Naveed Akhtar Qureshi

**Affiliations:** 1 School of Finance and Economics, Jiangsu University, Zhenjiang, China; 2 School of Business, Liaocheng University, Liaochengm Shandong, China; 3 Faculty of Commerce and Administration, Autonomous University of Tamaulipas, Ciudad Victoria, Mexico; 4 Department of Marketing and Entrepreneurship, Dhofar University, Salalah, Oman; 5 Department of Business Administration, Sukkur IBA University, Sukkur, Pakistan; Universite Ziane Achour - Djelfa, ALGERIA

## Abstract

Globally, healthcare workers (HCWs) are at high risk of acquiring Coronavirus infection. In addition, they are role models for the general public concerning attitudes towards the COVID-19 vaccine. In addition, they play a critical role in successfully promoting practices aiming to reduce the transmission of COVID-19 infection. Therefore, this study broadly based on the theory of planned behavior (TPB) explores the factors influencing HCWs’ intention to be vaccinated. An online survey was administered using Google Form to collect data from HCWs working in the public health sector of Pakistan. The sample included 813 participants, two-thirds were female, and one-third were male. In addition, 41.5% of them were aged between 26–35 years, 32.6% had master’s level education, 25% were nurses, and 57.7% of them were living in urban areas. Data analysis was run using Partial Least Squares Structural Equation Modeling (PLS-SEM). The research findings reveal the positive and significant effect of the TPB factors (attitude, subjective norms, and perceived behavioral control) and the extended factor of vaccine confidence on HCWs’ intention to be vaccinated. This study’s model explains 66.4% of variations in HCWs’ intention to be vaccinated.

## 1. Introduction

Globally, there have been over 515 million confirmed cases of COVID-19 and over 6.2 million deaths. Most of the confirmed cases were reported in the Europe, America, and South-East Asia region at 217, 153, and 57 million. Against this pandemic, over 1159 million COVID-19 vaccine doses have been administered [1]. Pakistan reported over 1.2 million confirmed cases, and 23.4% died [2]. To minimize the transmission of COVID-19 in late 2020 and early 2021, COVID-19 vaccines were approved for public use across the world [3].

COVID-19 Vaccines have been considered the most effective strategy to control the transmission of the virus among the general public [4], although preventive COVID-19 behaviors such as social distancing are also important [5–7]. Since it was approved in late 2020, most studies have highlighted the importance of COVID-19 vaccine hesitancy in the general public. Kukreti et al. [8] conducted a systematic review and meta-analysis among 11 countries (i.e., Turkey, Saudi Arabia, Jordan, Kuwait, Italy, China, Poland, UK, Greece, US, and France) on vaccine acceptance in the context of the general population and observed 73% highest willingness to get vaccinated among the countries with 1000–4000 cases per million population and conclude that hesitancy could be the major obstacle to the global effort to control the pandemic. Likewise, Yasmin et al. [9] conducted a systematic review regarding vaccine acceptance among the United States population and reported 12–91.4% overall vaccine acceptance. Besides, among the African/Black American there was increased unwillingness. In addition, age, education, income, race, and sex were having significant impacts on uptake of vaccine. However, Biswas et al. [3] and Kukreti et al. [10] calls for future studies to explore the factors influencing HCWs’ intentions toward the COVID-19 vaccine. Accordingly, Ahorsu et al. [11] and Ullah et al. [12] calls for studies on HCWs’ willingness and acceptance level. Furthermore, COVID-19 has subjected HCWs’ to a great extent of risk of infection through direct contact with patients, coupled with psychological stress and increased workload [13]. HCWs (i.e., doctors, nurses, pharmacists, community health workers, and midwives) are often prioritized to get vaccine [14], and their protection is essential for the country’s strategic response to combat COVID-19 [15].

Previously, studies identified the effect of the side effects of the vaccine, perceived benefits, age, insurance attitude, gender, marital status, region, monthly income, educational level, confidence in the government information, perceived susceptibility to COVID-19 and attitude towards the vaccine [16–18]. Despite the important role played by HCWs to persuade general public to get vaccine and spread the positive aspects of the vaccine, 42–61% of them have shown a lower level of COVID-19 vaccine acceptance [19]. Rad et al. [20] reported 32% vaccine hesitancy among HCWs’ in a cross-sectional study conducted in Iran. Rieger [21] observed that 71.4% of subjects in Germany were willing to accept the vaccine. There are several reasons for this lower level of acceptance or vaccine hesitancy, such as communication and media, religion, socioeconomic factors, risk perception, culture, and geographic barriers. 56% of HCWs’ reported COVID-19 vaccine hesitancy [22]. 76.4% HCWs were not sure about the safety, effectiveness, and efficacy of the COVID-19 vaccine [23].

Lack of information, confidence in the success ratio of vaccinations, lack of trust in vaccine manufacturing companies [24], concerns about safety/thinking that a vaccine produced in a rush is too dangerous, doubt about the efficiency of the vaccine [25,26], and some myths (i.e., vaccine makes people sexually abnormal, the embedding of a chip into the body, an illusion to target Islamic nations that the virus only affects older people and that the Coronavirus cannot harm Muslims, etc.), and cultural restrictions are significant barriers to be vaccinated in Pakistan [27,28]. In particular, 49% of the population and 43% of HCWs’ in Pakistan showed an unwillingness to accept the COVID-19 vaccine [29,30]. Although Pakistan received huge quantity of vaccine; (e.g., as of November 15, 2021, Pakistan administered 120 million COVID-19 doses), the intentions of HCWs’ to accept the vaccine and the determinants affecting their intention is still unexplored and requires more empirical studies [31]. The research insights from this part of the population would help the health sector and policy-makers improve COVID-19 vaccination acceptance, which would help control disease transmission. Therefore, this study aims to identify the factors influencing HCWs’ intentions of COVID-19 vaccination acceptance. In addition, this study used the theory of planned behavior (TPB) as it has been widely used in health-related intentions and behaviors. Also, this study also extended the explanatory power of the TPB by adding vaccine confidence as an additional variable.

## 2. Theoretical background and hypothesis development

This research is based on the theory of planned behavior (TPB) [32]. The TPB has been gradually used nowadays in studying distinct health-related intentions and actual behavior, including the uptake of the COVID-19 vaccine and people’s hesitancy to get vaccinated [33,34]. The TPB posits that an individual’s intentions predict an individual’s decision to engage in a specific behavior [35]. According to the TPB, three factors (i.e., personal attitudes, subjective norms, and perceived behavioral control) influence behavioral intention, and that behavior intention and perceived behavioral control influence actual behavior [36]. These components are described below

**Attitude:** refers to a person’s overall (favorable or unfavorable) evaluation of a particular behavior.
**Subjective norms:** refers to an individual’s belief about how others will think and approve or disapprove if she/he engages in the behavior.
**Perceived behavioral control** refers to an individual’s perception of the ease or difficulty of engaging in the behavioral intention.

Previously, some studies highlighted the importance of the TPB in the perspective of health-related behavior, and evidence is available that the TPB explains attitudes towards the COVID-19 vaccine, uptake intentions, and vaccine hesitancy [17,37]. In addition, it has been argued that the TPB is a valuable framework for explaining the COVID-19 vaccine intention [38,39]. In one meta-analysis study Xiao and Wong [40] using the TPB in the context of uptake and vaccine hesitancy, it was evidenced that the TPB explains the over 54% variance in intentions to get the vaccine. Likewise, Thaker and Ganchoudhuri [37] study evidenced that the TPB explains the 74% variation in an intention to get vaccinated. In addition, prior studies identified the link between perceived behavioral control and actual behavior, which has not been covered by Ajzen [32] old work. Later Ajzen [36] introduced the concept of perceived behavioral control. Such as Armitage and Conner [41] conducted a review study and observed that the perceived behavioral control construct is responsible for a significant variation in both intention and actual behavior. Accordingly, McDermott et al. [42] conducted a meta-analysis on the TPB and explored that the perceived behavioral control not only has a direct influence on both individual intentions but strengthens the direct effect of intention on actual behavior. In addition, it has been criticized that TPB failed to consider the impacts of behavior on cognition [43]. In response Sniehotta et al. [43] criticism, Ajzen [44] concluded that authors misunderstood the theory and misinterpreted the negative outcomes of poorly conducted research.

Previous scholars have added substantial contributions to the existing body of knowledge using TPB factors. However, other constructs can also be added to the TPB [36]. Therefore, this research used vaccine confidence in the TPB. Vaccine confidence refers to the trust that HCWs have in (a) the intentions of governments or policy-makers who make decisions regarding the vaccine, (b) the companies and professionals who are involved in the formation and delivery of the vaccine; and (c) the effectiveness and safety of the recommended vaccines [45]. Williams et al. [46] defined it as “a trust in the safety and efficacy of a vaccine”. Authors postulate that a low level of confidence leads to vaccine hesitancy and is observed as refusing and delaying, which is a major threat to the successful implementation of the COVID-19 promotion and campaign.

The COVID-19 vaccine is not trustworthy since there is skepticism and fear among the public regarding its effectiveness and safety [28]. There are undoubtedly other concerns, such as immediate and long-term side effects [18], lack of transparency in data sharing [47], and rapid development [48]. Moreover, it has been evidenced that vaccine confidence is directly related to uptake and vaccine acceptability [48]. Previously, [38,46], apart from the TPB theory constructs (i.e., attitude, subjective norms, and perceived behavioral control), used vaccine confidence as a different construct and evidenced the direct impact of vaccine confidence on an individual’s intention to get vaccinated. Similarly, scholars believe that vaccine confidence would improve the explanatory power of the TPB. Based on the above discussion, we hypothesize as:

H1: Attitude is a significant predictor of HCWs’ intention to get the COVID-19 vaccination.
H2: Subjective norms is a significant predictor of HCWs’ intention to get the COVID-19 vaccination.
H3: Perceived behavioral control is a significant predictor of HCWs’ intention to get the COVID-19 vaccination.
H4: Vaccine confidence is a significant predictor of HCWs’ intention to get the COVID-19 vaccination.

## 3. Materials and methods

### 3.1. Population and sample size

The study population includes the HCWs working in national hospitals in Sindh, Pakistan. By 2020, the total workforce of the national health sector was 454,496, which included 245,987 (doctors), 27,360 (dentists), 116,659 (nurses), 43,19 (midwives), and 21,361 (community health visitors) [49]. To collect the data from the HCWs, we approached the human resource/administrative staff of the hospitals after getting the personal information (name, occupation, emails, and numbers). We ensured both administrative staff and HCWs that their information would not be shared with anyone and would be used only for the study purpose. The minimum sample size was calculated using the following formula:

$$n=\frac{Z^{2}}{4d^{2}}=\frac{1.96^{2}}{4{\left(0.05\right)}^{2}}=\frac{3.8416}{0.01}=384.16\;\;\sim \;385$$

Where n = sample size, Z = level of confidence interval 95.0%, z = 1.96, d = tolerated margin of error 5% [50]. A minimum sample size of the study 385 HCWs was estimated. To generalize the results, we increased the sample size to 840. A web-based survey link was shared with 1500 HCWs from July 2021 and lasted for 15 days, which resulted in 840 responses, a response rate of 56%. Of them, 27 were removed from the analysis due to incomplete information, and 813 were included in the sample. Previously Shmueli [34] reported a sample size of 398, and Breslin et al. [33] had 439 participants from the general population. Therefore, based on the suggested calculation formula and relative to previous studies, we conclude that this research sample size is adequate.

### 3.2. Inclusion and exclusion

Those HCWs who (1) voluntarily agreed to participate in this survey; (2) worked in hospitals (3) were able to read and complete the questionnaire independently were included. While those who (1) responded being vaccinated (2) and responded as less than 18 years old; were excluded from the survey to ensure the reliability of data and encounter common method bias issues [51].

### 3.3. Recruitment

This study recruited a sample of HCWs working in national hospitals in Pakistan. Before the recruitment process was initiated, we contacted the hospital’s human resource/administrative staff for the necessary approval. Additionally, additionally, an ethical approval was obtained from the review board of the hospital and Jiangsu University, China, on the human participants. After getting a list of employees, we launched the web-based survey and disseminated the survey information to the potential population using their WhatsApp numbers, emails, and social media accounts. We ensured the participants that their participation would remain confidential, voluntary, and anonymous. In addition, their response will only be used for this study and will not be shared with any outside organizations. The study targeted all HCWs (i.e., doctors, dentists, nurses, midwives, and community health workers) since it is the suggested approach in recent COVID-19 studies [48].

### 3.4. Sampling and data collection

A cross-sectional web-based survey was conducted among 813 Pakistani HCWs. A convenient random sampling approach was employed to collect data through an online survey designed using Google Forms in both English and Urdu languages. Before sharing the survey link, two professors of Sukkur IBA University were involved to ensure the consistency of the constructs’ items. A survey link was shared through social media platforms (e.g., WhatsApp, Email, Facebook, etc.). In addition, there are several other benefits of web-based surveys, such as low administration and returning cost, interactive features, the anonymity of participants, and reach to a large audience. An online survey was employed to avoid common method bias, and respondents were allowed to submit a single response. For the counter-check, we recorded the respondents’ IP addresses. Moreover, data collection and electronic surveys are widely accepted and acknowledged in recent COVID-19-based studies [33,48,52]. For data collection purposes, a survey link was opened for 15 days in July 2021.

### 3.5. Survey design and measurements

This study used the theory of planned behavior (TPB) to determine the factors influencing HCWs’ intentions to accept the COVID-19 vaccination. This study used three TPB factors, namely attitudes, subjective norms, perceived behavioral control, and an additional factor, namely vaccine confidence, to improve the value of being vaccinated. As previously mentioned, this research used an online survey for data collection, which consists of 7 sections: The first section is comprised of demographic factors (i.e., gender, age, education, and occupation).

The second to seventh section consists of questions related to a specific construct. This study used a 5-point Likert scale [for (attitude, subjective norms, perceived behavioral control, and vaccine confidence responses to record, where 1 = strongly disagree, and 5 = strongly agree) and for (intention to get vaccinated 1 = very unlikely and 5 = very likely)] to record responses of HCWs. The attitude was measured using 5 questions adopted from Ogilvie et al. [53]. Subjective norms were assessed using 3 questions adapted from Chu and Lu [54]. Perceived behavioral control was assessed using 3 items adapted from Larson [53]. Vaccine confidence was measured using 2 questions adopted from Larson [55]. Intention to be vaccinated was assessed using 3 questions adapted from Chu and Liu [54].

### 3.6. Data analysis

#### 3.6.1 Univariate analysis

Table 1 demonstrates the univariate result analysis of the study. Table 1 reflects the overall score of the variables rated by participants following standard deviations. In addition, we used an independent test to evaluate the significance of the results criterion (p-value<0.05). Table 1 exhibits the scoring of HCWs related to attitude, subjective norms, perceived behavioral control, and vaccine confidence. For attitude scoring, 5 items were scored on a 5-point Likert scale with a maximum score of 25. Likewise, subjective norms, perceived behavioral control, vaccine confidence, and intention to be vaccinated scored 15, 15, 10, and 15, respectively (see Table 1).

**Table 1:** Univariate analysis of factors and HCWs’ intention to be vaccinated based on demographic factors.

Characteristics		Attitude score (25)	*p*-value	Subjective norm (15)	*p*-value	Perceived behavioral control (15)	*p*-value	Vaccine confidence (10)	*p*-value	Intention to be vaccinated (15)	*p*-value
Gender	Male	17.6 ± 0.26	<0.001	12.2 ± 0.16	<0.001	12.2 ± 0.16	<0.001	6.17 ± 0.14	0.667	10.9 ± 0.23	<0.001
	Female	16.1 ± 0.24		10.4 ± 0.13		10.7 ± 0.14		6.25 ± 0.10		9.13 ± 0.18	
Age	18–25	14.5 ± 0.31	<0.001	9.5 ± 0.19	<0.001	9.89 ± 0.20	<0.001	5.59 ± 0.22	0.011	7.56 ± 0.23	<0.001
	26–35	16.1 ± 0.28		11.0 ± 0.17		11.5 ± 0.17		6.34 ± 0.16		9.51 ± 0.24	
	36–45	20.6 ± 0.20		13.3 ± 0.10		13.4 ± 0.05		6.40 ± 0.13		13.5 ± 0.70	
	Over 45	18.6 ± 0.52		12.0 ± 0.28		11.2 ± 0.42		6.18 ± 0.24		11.3 ± 0.34	
Education	Basic/High school	16.4 ± 0.26	<0.001	10.9 ± 0.22	<0.001	11.2 ± 0.20	<0.001	5.98 ± 0.17	0.270	9.46 ± 0.27	<0.001
	Undergraduate	16.9 ± 0.28		11.0 ± 0.19		11.3 ± 0.21		6.28 ± 0.16		9.55 ± 0.27	
	Master’s	19.1 ± 0.37		12.4 ± 0.18		12.0 ± 0.17		6.27 ± 0.14		11.7 ± 0.25	
	Other	11.6 ± 0.42		8.75 ± 0.22		9.57 ± 0.34		6.5 ± 0.22		7.07 ± 0.35	
Occupation	Doctor	18.9 ± 0.31	<0.001	12.7 ± 0.14	<0.001	12.3 ± 0.19	<0.001	6.5 ± 0.18	0.017	12.1 ± 0.22	<0.001
	Dentist	18.5 ± 0.42		11.9 ± 0.24		12.2 ± 0.25		6.6 ± 0.20		10.8 ± 0.38	
	Nurse	15.9 ± 0.35		10.8 ± 0.21		11.4 ± 0.20		6.20 ± 0.17		9.5 ± 0.30	
	Midwives	14.4 ± 0.39		9.32 ± 0.24		9.74 ± 0.26		6.22 ± 0.21		7.59 ± 0.28	
	Community health worker	15.6 ± 0.5		10.3 ± 0.3		10.6 ± 0.31		5.74 ± 0.17		8.74 ± 0.40	

Table 1 reflects those males aged between 36 and 45 years, have a master’s level education, and as doctors, had a higher score on attitude, subjective norms, perceived behavioral control, and intention to be vaccinated. While, Females, youngsters (age between 15–25 years), others (education), and midwives (occupation) have a lower level of attitude, subjective norms, perceived behavioral control, and intention to be vaccinated. Furthermore, all of the demographics had positive and significant effects on study factors (attitude, subjective norms, perceived behavioral control, vaccine confidence, and intention to be vaccinated, except gender and education, has a non-significant (p-value>0.05) correlation with confidence in the vaccine (see Table 1).

#### 3.6.2 Partial Least Square—Structural Equation Modeling (PLS-SEM) analysis

Hair et al. [56] suggested the PLS-SEM model evaluate the reliability, consistency, and validity of the constructs. An assessment of the measurement model comprises outer loadings of factors for individual reliability, composite reliability (CR) to assess internal consistency, and average variance extracted (AVE) to assess validity. The individual item reliability Hair et al. [56] suggested that the value of factor loadings must be ≥0.7. In addition, regarding the Cronbach’s alpha (CA), it was suggested that it must be ≥0.7. The present study CA values retained between 0.899 and 0.960 (see Table 2). Related to internal consistency reliability, it was required that CR should be ≥0.7 (see Table 2). Regarding validity, it has been suggested that the AVE must be ≥0.5 [57]. The present study AVE value retained between 0.771 and 0.925 (see Table 2). This study used inner VIF to check CMB and multicollinearity issues suggested by Hair et al. [56] using PLS-SEM. Table 2 exhibits that CMB is not a concern since the VIF values ranged between 1.403 and 2.803, which is <3.33 acceptable threshold [56].

**Table 2:** Reliability and validity analysis (measurement model).

Constructs	Items	Loadings	CA	CR	AVE	Inner VIF
Attitude (A)	A1: A vaccine is essential to protect and be safe from deadly diseases.	0.880	0.926	0.944	0.771	1.752
	A2: A vaccine is vital to stop the COVID-19 pandemic.	0.904				
	A3: A COVID-19 vaccine should be beneficial for my health.	0.901				
	A4: A COVID-19 vaccine should be mandatory	0.877				
	A5: COVID-19 would be beneficial for HCWs irrespective of ages	0.827				
Subjective norms (SN)	SN1: Most people important to me think that I should receive the COVID-19 vaccine.	0.906	0.902	0.938	0.836	2.795
	SN2: I would feel pressure from those necessary to receive a COVID-19 vaccine.	0.910				
	SN3: Most of the people I care for will get a COVID-19 vaccine.	0.927				
Perceived behavioral control (PBC)	PBC1: It could be convenient to receive the COVID-19 vaccine.	0.931	0.912	0.944	0.850	2.590
	PBC2: I could quickly receive a COVID-19 vaccine if I wanted to.	0.914				
	PBC3: I am confident that I have significant knowledge of the COVID-19 vaccine.	0.921				
Vaccine confidence (VC)	VC1: Overall, I am confident that public authorities decide in the best interest of HCWs to protect them from infection.	0.948	0.899	0.952	0.908	1.142
	VC2: I am confident that the COVID-19 vaccine is effective and safe.	0.958				
Intention to be vaccinated (ITBV)	ITBV1: I am trying to get the COVID-19 vaccine	0.967	0.960	0.974	0.925	
	ITBV2: I am willing to get vaccinated to avoid spreading the virus.	0.961				
	ITBV3: I am willing to get vaccinated if my professional prescribes me.	0.954				

Table 3 reflects that HTMT values are below the 0.85 acceptable standards [56,58] and the inter-correlations among the variables. Hence, discriminant validity was confirmed for the variables included in the model.

**Table 3:** Heterotrait-Monotrait ratio.

Constructs	A	ITBV	PBC	SN	VC
Attitude (A)					
Intention to be vaccinated (ITBV)	0.700				
Perceived behavior control (PBC)	0.630	0.747			
Subjective norm (SN)	0.695	0.799	0.842		
Vaccine confidence (VC)	0.214	0.109	0.384	0.333	

## 4. Results and discussions

### 4.1. Descriptive results

Table 4 reflects that out of 813 HCWs 65.4% were female, and 24.6% were male. Participants 18–25 (256), 26–35 (335), 36–45 (130), and older than 45 (91) accounted for 31.5, 41.2, 16.0, and 11.3%, respectively. Regarding education basic/high school (214), undergraduate (221), masters (265), and other (113) accounted for 26.3, 27.2, 32.6, and 13.9%, respectively. Regarding the occupation status, doctors (186), dentists (115), nurses (206), and midwives (125) accounted for 22.9, 14.1, 25.3, and 15.4%, respectively.

**Table 4:** Respondents’ information.

Variables		Frequency	Percentage
Gender	Female	532	65.4
	Male	281	34.6
Age (years)	18–25	256	31.5
	26–35	335	41.2
	36–45	130	16.0
	Over 45	91	11.3
Education	Basic/high school	214	26.3
	Undergraduate	221	27.2
	Master’s	265	32.6
	Other	113	13.9
Occupation	Doctor	186	22.9
	Dentists	115	14.1
	Nurse	206	25.3
	Midwives	125	15.4
	Community health worker	181	22.3

Table 5 reveals several participants and proportions in the following format n(%). Regarding attitude, nearly 60% (43.9 + 15.9) of HCWs have shown a willingness to be vaccinated, whereas 40% (12.4 + 11.2 + 16.6) reported disagreement or neutral response. In addition to this, 55% (41.7 + 13.3) believe that vaccines can stop the pandemic, while 45% were not sure or have shown a neutral attitude about the efficiency of the vaccine. Furthermore, over half of the participants were not clear about the benefits of vaccination. However, nearly two-thirds of them reported that it is mandatory for public health, and nearly half of them have stated that the COVID-19 vaccine should be beneficial for HCWs irrespective of age.

Regarding the subjective norms, nearly two-thirds of them reported that they should be vaccinated, had pressure to be vaccinated, and assumed their loved ones should be vaccinated. Regarding perceived behavioral control, approximately three-quarters of them stated that it is convenient and easy to get the vaccine, and they have significant knowledge now about vaccination. Regarding confidence toward the vaccine’s usefulness, 43.2% and 42.9% stated that vaccination is essential for HCWs and the public, primarily to protect themselves (see Table 5).

**Table 5:** HCWs response against each individual construct item.

Construct	Items	SD* n (%)	D* n (%)	N* n (%)	A* n (%)	SA* n (%)
Attitude (A)	A1: A vaccine is essential to protect and be safe from deadly diseases.	101 (12.4)	91 (11.2)	135 (16.6)	357 (43.9)	129 (15.9)
	A2: A vaccine is vital to stop the COVID-19 pandemic.	79 (9.7)	142 (17.5)	145 (17.8)	339 (41.7)	108 (13.3)
	A3: A COVID-19 vaccine should be beneficial for my health.	116 (14.3)	114 (14.0)	231 (28.4)	192 (23.6)	160 (19.7)
	A4: A COVID-19 vaccine should be mandatory	20 (2.5)	132 (16.2)	147 (18.1)	451 (55.5)	63 (7.7)
	A5: COVID-19 would be beneficial for HCWs irrespective of ages	79 (9.7)	202 (24.8)	151 (18.6)	203 (25.0)	178 (21.9)
Subjective norms (SN)	SN1: Most people important to me think that I should receive the COVID-19 vaccine.	35 (4.3)	109 (13.4)	147 (18.1)	354 (43.5)	168 (20.7)
	SN2: I would feel pressure from those necessary to receive a COVID-19 vaccine.	55 (6.8)	83 (10.2)	157 (19.3)	345 (42.4)	173 (21.3)
	SN3: Most of the people I care for will get the COVID-19 vaccine.	35 (4.3)	135 (16.6)	98 (12.1)	243 (29.9)	302 (37.1)
Perceived behavioral control (PBC)	PBC1: It could be convenient to receive the COVID-19 vaccine.	57 (7.0)	88 (10.8)	93 (11.4)	415 (51.0)	160 (19.7)
	PBC2: I could quickly receive a COVID-19 vaccine if I wanted to.	68 (8.4)	75 (9.2)	67 (8.2)	306 (37.6)	297 (36.5)
	PBC3: I am confident that I have significant knowledge of the COVID-19 vaccine.	36 (4.4)	117 (14.4)	96 (11.8)	326 (40.1)	238 (29.3)
Vaccine confidence	VC1: Overall, I am confident that public authorities decide in the best interest of HCWs to protect them from infection.	113 (13.9)	166 (20.4)	183 (22.5)	250 (30.8)	101 (12.4)
	VC2: I am confident that the COVID-19 vaccine is effective and safe.	84 (10.3)	226 (27.8)	154 (18.9)	178 (21.9)	171 (21.0)
Intention to be vaccinated (ITBV)	ITBV1: I am trying to get the COVID-19 vaccine	130 (16.0)	186 (22.9)	24 (3.0)	293 (36.0)	180 (22.1)
	ITBV2: I am willing to get vaccinated to avoid spreading the virus.	173 (21.3)	145 (17.8)	26 (3.2)	296 (36.4)	173 (21.3)
	ITBV3: I am willing to get vaccinated if my professional prescribes me.	151 (18.6)	167 (20.5)	21 (2.6)	224 (27.6)	250 (30.8)

Note

*: *SD = strongly disagree; D = disagree; N = neutral; A = agree; SA = strongly agree*.

### 4.2. Hypotheses results

We used 5,000 bootstraps recommended by Hair et al. [56] using SmartPLS 4.0 and 813 cases to determine the effects of TPB and vaccine confidence factors that impact their intention to be vaccinated. Hypothesis testing using the level of significance is the first measure for assessing the structural model. Table 6 shows that all hypotheses were supported, given that the p-value <0.05. Coefficients of determination (R^2^) was the second measure. R^2^ values of 0.25, 0.5, and 0.75 can be considered weak, moderate, and substantial, respectively [56]. This study retained an R^2^ value of 0.669, indicating that 66.9% of the changes in intentions to be vaccinated occurred because of four factors, namely attitude, subjective norms, perceived behavioral control, and confidence in the vaccine (see Table 6).

**Table 6:** Hypothesis testing and strength of a model.

Hypothesis	Relationship	Path coefficient	SD	*t*-value	Decision
*H1*	Attitude→ Intention to be vaccinated	0.267	0.027	9.710**	Supported
*H2*	Subjective norms→ Intention to be vaccinated	0.398	0.038	10.405**	Supported
*H3*	Perceived behavioral control→ Intention to be vaccinated	0.300	0.035	8.680**	Supported
*H4*	Vaccine confidence → Intention to be vaccinated	0.175	0.021	8.508**	Supported

Notes:

Critical values.

*t-value>1.96 (*p*<0.05)

**(*p*<0.001).

*R*^*2*^ (Intention to be vaccinated) = 0.664.

Goodness of fit→ SRMR = 0.041, Chi–Square = 1,413.794, NFI = 0.888.

Regarding the goodness of the fit index, we used Standardized Root Mean Square Residual (SRMSR). SRMSR is an absolute measure of fit: a value of zero indicates a perfect fit, and a value less than 0.08 is considered a good fit [56]. In addition, NFI (normed fit index) is suggested to be greater than 0.7 and closer to 1 [59]. This study’s NFI value of 0.888 falls within the acceptable threshold [60,61]. Table 6 demonstrates the study’s adequate goodness of fit.

We observed the attitude toward the COVID-19 vaccine has a positive and significant effect on HCWs’ intention to be vaccinated (β = 0.255, t = 8.980, p = 0.000); thus, we supported *H1*. Furthermore, we evidenced the positive and significant effect of subjective norms on HCWs’ intentions to be vaccinated (β = 0.381, t = 9.621, p = 0.000); therefore, *H2* was supported. In addition, we find the positive and significant impact of perceived behavioral control on intention to be vaccinated (β = 0.266, t = 7.684, p = 0.000); therefore, *H3* is supported. Finally, we observed the positive and significant effect of vaccine confidence on intention to be vaccinated (β = 0.213, t = 8.387, p = 0.000); therefore, we have supported *H4* (See Table 6).

### 4.3 Discussion of results

Globally, the public and HCWs consider the COVID-19 pandemic a severe issue. The majority of respondents have highlighted the significant importance of the COVID-19 vaccine and think people must receive the vaccine and protect themselves. However, due to a lack of trust in the vaccine manufacturers, cultural and religious constraints, safety concerns, and unsupported myths, a lower level of intention was reported to accept vaccines [19,22]. In this context, to the best of the authors’ knowledge, this is the first time to explore the factors influencing HCWs’ intentions toward the COVID-19 vaccine’s acceptance in Pakistan. This study evidenced that 66.4% of the variation among HCWs’ intention to be vaccinated was explained by attitude, subjective norms, perceived behavioral control, and vaccine confidence. This finding aligns with previous studies of Dror et al. [62], who reported a vaccine acceptance rate of 75% in Israel, and [38], who evidenced a 65% acceptance rate among Indian populations.

Regarding the effects of age and education. Our study findings exhibit those male participants aged 26–35 years with a master’s level of education had a higher level of vaccine acceptance, similar to the previous work of Ogilvie et al. [34] and Shumeli [53]. It is reasonable to have a higher acceptance rate among male, well-educated, and aged people. It is assumed that they will have more knowledge and information about the COVID-19 vaccine relative to females, those less educated, and those aged below 30 years [50]. Concerning occupations, doctors, dentists, and nurses have a higher acceptance rate of the COVID-19 vaccine, as they have more contact with patients’ relatives, midwives, and community health workers. In addition, midwives and community health workers have a lower level of education relative to doctors, dentists, and nurses in Pakistan. The outcome of this part of the study is consistent with the prior work of Al-Sanafi and Sallam [63]. Furthermore, the findings evidenced that demographic constructs (i.e., gender, age, education, and occupation) significantly influenced participants’ intentions to be vaccinated. These findings are also consistent with the prior work of Li et al. [64] and Yang et al [65].

The findings further evidence that the TPB factors were found to have a positive and significant effect on HCWs’ intention toward the COVID-19 vaccine. The relative contribution of subjective norms and perceived behavioral control is larger than attitude. This finding is supported by the previous work of Shumeli [34]. Subjective norms emerged as the most influential factors, followed by attitude, to receive the COVID-19 vaccine [54]. Subjective has a larger effect because participants care more about the families and their opinions. Specifically, this study evidenced that if there is a single unit change in HCWs positive attitude towards COVID-19 vaccination, it will increase by 25.5% the intention to get vaccinated (Table 6). In addition, approximately 60% of participants showed willingness, over half of them believe that it can stop the pandemic, and nearly 64% reported it is beneficial for all ages of HCWs (Table 5). This finding is in line with the prior work of Li et al. [23] and Pogue et al. [66], who evidenced that, in general, HCWs hold a positive attitude towards the COVID-19 vaccination.

Regarding the second TPB factor, findings suggest that subjective norms have a most robust effect on the participant’s acceptance of the vaccine. The results infer that a single unit increase in subjective norms leads to a 38.1% change in HCWs’ intentions to get the COVID-19 vaccine. In addition, 64.2, 63.7, and 67.0% reported that they should be vaccinated because of their loved ones, they have pressure from families, and their loved ones will receive the vaccine, respectively. These findings infer that HCWs are greatly influenced by their loved ones to get vaccinated. This finding of the study is consistent with the previous work of Ogilvie et al. [53].

This study evidenced that perceived behavioral control has the second most significant influence on HCWs’ intention to be vaccinated. This finding reveals that a single unit change in perceived behavioral control leads to a 26.6% change in their intention to be vaccinated. In addition, the results demonstrate that HCWs have a significant perceived behavioral control as 70.1, 74.1, and 69.4% it is convenient and easy to receive, and they have significant knowledge about the COVID-19 vaccine, respectively. The study’s findings are in line with the previous work of Breslin et al. [33]. In addition, this study also supported the previous work of Husain et al. [38], who evidenced the significant effect of perceived behavioral control on the Indian population toward acceptance of the COVID-19 vaccine.

Finally, this study evidenced a positive and significant impact of vaccine confidence on HCWs’ intention to receive vaccines. This study’s results state that if confidence in a vaccine increases by 1 unit, the intention to get vaccinated will increase by 21.3%. This research finding infers that participants’ intention to get a vaccine increase when they had confidence in the health department and the effectiveness of the COVID-19 vaccine. These findings are consistent with the prior work of Larson [55].

## 5. Conclusion

Globally, the COVID-19 pandemic has disrupted world economies and our ways of living and has wreaked havoc on millions of individuals’ lives, and resulted in over 5.1 million deaths worldwide. As Pakistan is one of the worst-hit countries by COVID-19, there is a considerable need for mass vaccination, and its acceptance rate must increase. This research offers up-to-date data on the HCWs’ intention to receive the COVID-19 vaccine. The current research explored the effect of attitude, subjective norms, perceived behavioral control, and vaccine confidence on HCWs’ intention to be vaccinated in the public sector of Pakistan. To the best of our knowledge, this is the first attempt in Pakistan for this type of research within the public health department. In addition, a proposed framework of the study was drawn from the TPB, using PLS-SEM techniques, and an analysis was performed. Our results recognized that although most HCWs want to receive the vaccination, their intentions differ based on their demographics (i.e., gender, age, education, and occupation) and the TPB factors of attitude, subjective norms, and perceived behavioral control as well as the extended factor of vaccine confidence.

### 5.1. Implications

The present study highlighted that the TPB is an effective model in explaining and predicting HCWs’ intentions to get vaccinated. In addition, along with the TPB factors, vaccine confidence has also had a significant influence on the intention to receive vaccines. To enlarge the HCWs positive attitude toward getting vaccinated, the government and health department requires to run a campaign to improve the importance of getting vaccinated. In addition, families, friends, and relatives also be motivated via different forms or advertisements to motivate and increase the level of responsibility among HCWs to get vaccinated. Moreover, HCWs can easily get the vaccine because they are on a high-priority level and at great risk of infection. However, they are unsure about how effective the vaccine is, which decreases their confidence level. The findings suggest that public and private institutes cumulatively enlarge the awareness and positive aspects of vaccinating. In the existing literature, there is also substantial evidence that intention gradually results in actual behavior. Thus, the result of the present study can be employed to develop strategies regarding the successful development of vaccination programs.

Furthermore, this study helps the health department increase knowledge and awareness about the benefits of the COVID-19 vaccination; therefore, the postponement of being vaccinated may be decreased, and the low level of confidence in getting vaccinated may improve. In addition, the health department arranges programs to remove the myths and misinformation regarding the adverse side effects of vaccination among the HCWs. This study’s results are crucial for the government and policy-makers and can provide a better guide to vaccination compliance. In particular, efforts must be exerted to target females (i.e., midwives and community health workers), non-academics, and those who choose to follow a more religious interpretation of events and/or are under 18 years.

### 5.2. Limitations and future research direction

This study also has several limitations. First, the present study sample includes HCWs of public hospitals; therefore, this study’s results cannot be generalized to encompass all HCWs working in private hospitals. Thus, it is suggested that future researchers can replicate the model in private hospitals or with mixed samples from both populations. Secondly, this study included the 3 TPB and vaccine confidence impacts on intention to be vaccinated. However, the TPB does not include other factors such as perceived trust and moral responsibility, etc. Thus, it is proposed that future scholars investigate and incorporate these other factors’ impacts because trust could help to build the attitude and moral responsibility realizes the participants to prevent society there is need to get vaccinated. Thirdly, our study did not examine the actual behavior of HCWs in getting vaccinated. In addition, the TPB did not address the relationship between intention and behavioral action, nor did it include economic and environmental factors which can influence an individual’s intention to perform a specific behavior. Also, our research did not explore the relationship between perceived behavioral control and actual behavior. Therefore, it is suggested that future scholars examine the relationship between the intention to be vaccinated, actual behavior, perceived behavioral control, and actual behavior. Fourthly, it is suggested that as this study was conducted in developing countries, the results may be implemented in other developing countries, for instance, Bangladesh, India, and Iran. However, different cultures must also be considered an essential factor when comparing countries. Finally, sampling techniques, cross-sectional nature, and data collection tools may have created limitations. Thus, it is recommended that future research should collect data through an offline/field survey to validate the results.

## Supporting information

S1 File (RAR)

S2 File (DOCX)

S3 File (DOCX)
