# Supplementary material for: Understanding factors influencing healthcare workers’ intention towards the COVID-19 vaccine
Source: PLoS One. 2023 Jul 27;18(7):e0286794. doi: 10.1371/journal.pone.0286794 (PMC10374054; doi:10.1371/journal.pone.0286794)
Supplement: S3 File — (DOCX) [file pone.0286794.s003.docx]

$$n=\frac{Z^{2}}{4d^{2}}=\frac{{1.96}^{2}}{4{(0.05)}^{2}}=\frac{3.8416}{0.01}=384.16 ͠ 385$$

**Informed Consent Statement:** The participant's consent to participate in this study.

**Questionnaire**

**Demographics information**

*1- Mention your gender below*

*Male Female*

*2- Select your age group (years)*

18 – 25 26 – 35 36 – 45 Over 45

*3- Indicate your education level*

*Basic/high school Undergraduate Master’s Other*

*4- Indicate your position in the firm*

Doctor Dentists Nurse Midwives

Community health worker

**Variables statements**

Please indicate your opinion by marking the appropriate box on the five-point Likert scale where (*1 = Strongly Disagree, 2 = Disagree, 3= Neutral, 4 = Agree, 5 = Strongly Agree*)

| Attitude (A) | **Statements** | **1** | **2** | **3** | **4** | **5** |
| --- | --- | --- | --- | --- | --- | --- |
|  | A vaccine is essential to protect and be safe from deadly diseases. |  |  |  |  |  |
|  | A vaccine is vital to stop the COVID-19 pandemic. |  |  |  |  |  |
|  | A COVID-19 vaccine should be beneficial for my health. |  |  |  |  |  |
|  | A COVID-19 vaccine should be mandatory |  |  |  |  |  |
|  | COVID-19 would be beneficial for HCWs irrespective of ages |  |  |  |  |  |
| Subjective norms (SN) | Most people important to me think that I should receive the COVID-19 vaccine. |  |  |  |  |  |
|  | I would feel pressure from those necessary to receive a COVID-19 vaccine. |  |  |  |  |  |
|  | Most of the people I care for will get a COVID-19 vaccine. |  |  |  |  |  |
| Perceived behavioral control (PBC) | It could be convenient to receive the COVID-19 vaccine. |  |  |  |  |  |
|  | I could quickly receive a COVID-19 vaccine if I wanted to. |  |  |  |  |  |
|  | I am confident that I have significant knowledge of the COVID-19 vaccine. |  |  |  |  |  |
| Vaccine confidence (VC) | Overall, I am confident that public authorities decide in the best interest of HCWs to protect them from infection. |  |  |  |  |  |
|  | I am confident that the COVID-19 vaccine is effective and safe. |  |  |  |  |  |
| Intention to be vaccinated (ITBV) | I am trying to get the COVID-19 vaccine |  |  |  |  |  |
|  | I am willing to get vaccinated to avoid spreading the virus. |  |  |  |  |  |
|  | I am willing to get vaccinated if my professional prescribes me. |  |  |  |  |  |
